# Longitudinal Associations Between Trauma Exposure and Executive Functions in Children: Findings from a Dutch Birth Cohort Study

**DOI:** 10.1007/s10802-021-00847-4

**Published:** 2021-09-05

**Authors:** R. Op den Kelder, A. L. Van den Akker, J. B. M. Ensink, H. M. Geurts, G. Overbeek, S. R. de Rooij, T. G. M. Vrijkotte, R. J. L. Lindauer

**Affiliations:** 1 Research Institute of Child Development and Education, University of Amsterdam, Amsterdam, The Netherlands; 2 Levvel Academic Center for Child and Adolescent Psychiatry, Amsterdam, The Netherlands; 3 Department of Child and Adolescent Psychiatry, Amsterdam UMC, Location AMC, University of Amsterdam, Amsterdam, The Netherlands; 4 Research Institute of Child Development and Education/Research Priority Area YIELD, University of Amsterdam, Amsterdam, The Netherlands; 5 Department of Psychology (Brain and Cognition)/ Research Priority Area YIELD, University of Amsterdam, Amsterdam, The Netherlands; 6 Department of Public Health, Amsterdam Public Health Research Institute, Amsterdam UMC, University of Amsterdam, Amsterdam, The Netherlands; 7 Department of Clinical Epidemiology, Biostatistics and Bio-Informatics, Amsterdam UMC, University of Amsterdam, Amsterdam, The Netherlands

**Keywords:** Trauma exposure, Children, Executive functioning, Structural equation modeling, Parenting behavior

## Abstract

This study is the first to distinguish two possible predictive directions between trauma exposure and executive functioning in children in a community sample. The sample consists of 1006 children from two time points with a seven years’ time interval of a longitudinal Dutch birth cohort study, the ABCD-study (Van Eijsden et al., 2011). We analyzed the longitudinal associations between trauma exposure and executive functioning using structural equation modeling. The results demonstrated that (after controlling for prenatal substance exposure and mothers’ educational level) trauma exposure before age 5 is predictive of poorer executive functioning at age 12 and trauma exposure between age 6 and 12. However, the association between executive functioning at age 5 and trauma exposure between age 6 and 12 was not statistically significant. Our results indicate that early life trauma exposure has a long term impact on later executive functioning and not the other way around. On top of that, trauma exposure seems to accumulate across childhood when children are exposed to a traumatic event before the age of 5. When looking at the potential moderating role of parenting behavior we found no evidence for such a moderating effect of parenting behavior. Our findings showed that children exposed to trauma early in life may experience problems in executive functioning later in life and they seem at higher risk for cumulative trauma exposure. Clinical practice should take this into account in both the way they provide (early) mental health care and in prevention and recognition of early trauma exposure.

## Introduction

Approximately two-thirds of youth across the globe are exposed to traumatic events before they are sixteen (Copeland et al., 2007; McLaughlin et al., 2013). According to the Diagnostic and Statistical Manual of Mental Disorders 5, trauma exposure is defined as exposure to an actual or threatened death, serious injury or sexual violation (American Psychiatric Association, 2000, 2013). A person is exposed to a traumatic event when the person directly experiences the traumatic event, witnesses the event in person or learns that the event occurred to a close family member or close friend. Examples of possible traumatic events are child sexual abuse, traffic accidents, physical abuse, natural disasters, and war-related experiences. Trauma-exposed youth are at heightened risk for the development of various emotional, behavioral, and physical health problems in both the short and long term (Fowler et al., 2009; Norman et al., 2012; Wegman & Stetler, 2009). As there are serious consequences of trauma exposure in childhood on both the individual and societal level, it is important to both prevent trauma exposure and the development of problems after trauma exposure.

A substantial body of research has shown that trauma-exposed youth do not only experience emotional and behavioral adjustment difficulties, but that they also experience problems with basic cognitive functions. A frequently reported finding in the cognition research field is that trauma-exposed youth show problems in executive functioning (Malarbi et al., 2017; Op den Kelder et al., 2018). Executive functions are a set of cognitive skills that are needed for goal-directed behavior. These functions play a crucial role in an individuals’ daily functioning (Diamond, 2013; Goldstein et al., 2014). Children with executive functioning problems experience difficulties with (1) dealing flexibly with and adapting to new situations, rules, and perspectives, (2) inhibiting automatic responses, thoughts, feelings, and (3) simultaneously storing and manipulating incoming information (Diamond, 2013; Miyake et al., 2000). Poor executive functioning can have serious impact on the quality of life (Brown & Landgraf, 2010), increases the risk for obesity (Miller, Lee, & Lumeng 2014) and substance abuse (Kim-Spoon et al., 2017), and is associated with lower academic achievements and more difficulties in finding and maintaining a job (Diamond, 2013).

Trauma exposure may not only impact the development of executive functions in children, problems in executive functions could predate and increase the risk for subsequent trauma exposure. There could be different explanations for this direction from early executive functioning to later trauma exposure. First, children with lower executive functions have more behavioral problems which increases the risk for interpersonal trauma such as child abuse or community violence as they are more difficult to handle at home or at school. Weaker executive functioning in preschool children has been associated with more behavioral problems according to a meta-analysis based on 22 studies (Schoemaker et al., 2013). A prospective study among 69 five year-old children found that early inhibitory control predicted behavioral problems at six years old (Quistberg & Mueller, 2020). Another study among elementary school children with oppositional/conduct problems showed that these children were at increased risk for peer victimization (Ter-Stepanian et al., 2019). Another potential pathway through which problems in executive functioning could lead to trauma exposure is that children with lower executive functioning are more vulnerable and therefore at higher risk of victimization or exploitation by adults. For example, a study among 92 adolescents found that children with lower executive functioning had a higher risk of being victimized by their peers (Kloosterman et al., 2014). Another study among 1377 children showed that inhibition at age 4 was associated with a higher risk of being a victim of bullying (Verlinden et al., 2014), which can also be considered traumatic in cases of physical threat or harm.

Although several longitudinal studies investigated the associations between early trauma and later executive functioning (Bos et al., 2009; McDermott et al., 2012, 2013), these studies did not make any attempt to control for early executive functioning making it impossible to draw conclusions about the direction of effects between trauma exposure and executive functioning. However, two longitudinal studies on trauma exposure in relation to intelligence and academic skills (which is closely related to executive functioning) (van Aken et al., 2016), did take early cognitive functioning into account. A longitudinal study among 206 children found that children exposed to interpersonal trauma exposure between birth and 64 months had lower scores on cognitive outcomes such as memory learning, problem solving, abstract thinking, and mathematical concept formation at 24 months (Bayley Mental Development Scale). These children also had lower scores on cognitive outcomes on subtests of the Wechsler’s preschool intelligence scale (Block Design, Vocabulary, and Animal House) at 64 months (Enlow et al., 2012). Another longitudinal birth cohort study among 8928 participants showed long term effects of childhood neglect on reading, mathematics, and general ability tests at age 7, 11, 16, and 50 year old when taking into account the earlier cognitive scores using multivariate response modeling (Geoffroy et al., 2016). Based on earlier research, we assume that trauma exposure is predictive of executive functioning, but to date, there is no longitudinal research confirming this link with executive functions specifically. Additionally, to date, there is no longitudinal research examining the possible predictive relationship of early executive functioning to later trauma exposure. This knowledge gap highlights the importance to investigate whether early trauma exposure predicts problems in executive functions and/or whether problems in executive functions predicts later trauma exposure.

### Parenting Behavior

Especially in childhood it is important to take the child’s context in consideration. The relationship between trauma exposure and executive functioning could be influenced by the child’s family environment. Specifically, parenting behaviors could function as putative moderators (i.e., buffers or exacerbators). Three parenting styles are mostly distinguished: authoritative, authoritarian, and permissive parenting (Baumrind, 1971). Parenting styles are about attitudes, values, beliefs about children’s nature, and specific parenting practices. The authoritative parenting style reflects high responsiveness and control, while the authoritarian parenting style reflects high control, but low responsiveness, and permissive parenting style reflects highly responsiveness but little control (Baumrind, 1971; Darling & Steinberg, 1993; Steinberg et al., 1992). It is possible that these variations in the degree of responsiveness and parental control moderates in the association between trauma exposure and executive functioning.

First, high levels of responsiveness and control in parenting behavior are known to be associated with adaptive coping styles and resilience in children (e.g. Afifi & MacMillan, 2011; Lind et al., 2018). Children who grow up in a parenting context that is more sensitive and supportive, might be better equipped to cope with trauma exposure as their parents might be more aware of the potential impact of trauma exposure, which in turn may lead parents to give more support and help their children when needed. As far as we know, to date, there is no research investigating parenting in the context of early trauma exposure and later executive functioning. Based on the resilience framework, the assumption is that parents who are more regulating and responsive are better in enhancing resilience for adverse life events. Resilience is defined as a good adaptation in a dynamic system after disturbances that are a threat to the system, its viability or development (Masten, 2014). Although not specifically focused on executive functioning, previous studies have shown that parenting behaviors might influence how much children are impacted by a traumatic event more generally. For instance, a large cross-sectional study among 5765 adolescents showed that an authoritarian parenting style had a significant negative effect on children’s resilience, indicating that they are more impacted by a traumatic event (Zhai et al., 2015). Another cross-sectional study among 358 school-aged children showed that children from parents who provided relatively little care to their children had a relatively high risk of developing internalizing problems after experiencing mass trauma (Sriskandarajah et al., 2015). More specifically, in a sample of 74 children that grew up in a household with intimate partner violence, positive parenting practices of the mother were related to a higher level of executive functioning (Samuelson et al., 2012). In sum, authoritative parenting behavior might buffer the impact of trauma exposure, and in turn, protect children from developing executive functioning problems. In contrast, authoritarian or permissive parenting behaviors could strengthen the negative relationship between trauma exposure and later executive functioning.

Parenting behavior could also moderate the relationship between early executive functioning and later trauma exposure. For example, responsive parents may be more likely to recognize that their child has problems with executive functioning and consequently may be more inclined to help children to regulate and structure their environment to a greater extent, which diminishes the risk for trauma exposure for their children. In a related vein, permissive parents will not likely regulate and control the child’s environment, sometimes children who are low in executive functioning need more. In other words, when children have lower executive functioning and parents are not able to guide their child in a responsive way, the relationship between executive functioning and later trauma exposure could be stronger. Previous research among 169 children aged 9 to 13 years has shown that parenting with high involvement and responsibility was associated with better performances on executive functioning tasks (Sosic-Vasic et al., 2017). Another study among 82 children and adolescents with ADHD or ASD showed that authoritarian and permissive parenting styles were associated with poorer executive functioning (Hutchison et al., 2016).There is no previous research that investigated parenting in the light of early executive functioning and later trauma exposure or broader risks for children. Therefore, we are in need of longitudinal research for more stringent and temporally informative tests about the possible moderating role of parenting on the relationship between trauma exposure and executive functions as well as the other way around.

Besides parenting behaviors, other factors may impact the bidirectional relationship between trauma exposure and executive functioning. First of all, a review has shown that children who have been exposed to both prenatal maternal alcohol use and a traumatic event are more likely to show deficits in attention, memory, intelligence and increased behavioral problems (Price et al., 2017). Second, prenatal exposure to cannabis or cigarettes is also related with problems in executive functioning in both young and older children (Fried & Smith, 2001; Micalizzi & Knopik, 2018; Noland et al., 2003; Richardson et al., 2002). Third, previous research suggested that parents’ educational level is negatively associated with executive functioning (Ardila et al., 2010) and that a low educational level is a risk factor for trauma exposure (Brattström et al., 2015). Furthermore, a systematic review showed that prenatal exposure to drugs, alcohol or tobacco and educational level influences the relationship between parenting practices and executive functioning in childhood (Fay-Stammbach et al., 2014). Therefore, each of these factors were included in our analytic model to control for them in the bidirectional associations between trauma exposure and executive functioning.

**Fig. 1:**
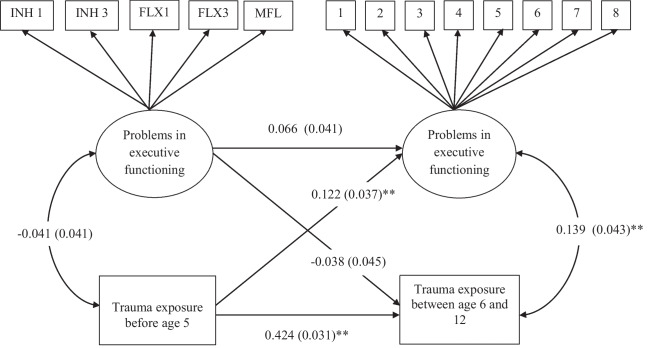
Model of the longitudinal and bidirectional associations between trauma exposure and executive functioning Path coefficients are standardized. Variables: INH1 = inhibition 1, INH3 = inhibition 3, FLX1 = flexibility 1, FLX3 = Flexibility 3, MFL = motor flexibility. Observed variables for factor of executive functioning at age 12 represent the sumscore of three items of each subscale of the Behavior Rating Inventory of Executive Functioning (BRIEF) **p < *0.001*

In our study, our aim was to investigate the longitudinal and bidirectional associations between trauma exposure and executive functioning as depicted in Fig. 1. We hypothesized that there would be a longitudinal relationship between early trauma exposure and later executive functioning and between early executive functioning and later trauma exposure. We also hypothesized that authoritarian and permissive parenting would strengthen these longitudinal associations, and that an authoritative parenting behavior would decrease the strength of these longitudinal associations.

## Method

### Study Population

The present study is part of the Amsterdam Born Children and their Development (ABCD) study (Van Eijsden et al., 2011). The ABCD study is an ongoing prospective birth cohort study among 8000 children, followed since pregnancy. The medical ethical committee of the Academic Medical Center of Amsterdam approved the ABCD cohort study (NL53940.018.15, study number: 2015_154).

From January 2003 until March 2004, all pregnant women in Amsterdam were asked to participate in the ABCD study during their first prenatal care visit. In total, 8266 pregnant women filled out the questionnaire and 6735 (81%) gave permission for follow-up. After 5 years (wave 3), 6161 mothers were retrieved and 4488 mothers reported on their children’s health. At age 11–12 (wave 4), 2997 mothers reported about their children’s health and 1006 children participated in physical examinations and interviews. We used information of these 1006 children that participated in both wave 3 and 4. We will further address these measurement waves as time point 1 (T1 = wave 3) and time point 2 (T2 = wave 4). The other waves primarily focused on physical health of the pregnant mother and the newborn child and thus could not be used for our study purposes.

The 1006 children who participated at T1 and T2 had a mean age of 5.1 (*SD* 0.23; range 5.0–7.2*)* at T1 and a mean age of 11.8 at T2 (*SD* 0.37; range 10.5—12.9). Of these participants, 49.0% were girls. Ethnicity was defined by the country of birth of the mother (Menting et al., 2018; Van Eijsden et al., 2011) and was divided into Dutch (82%) or non-Dutch (18%). Non-Dutch included the following ethnicities: Surinam (2.9%), Antilleans (1.0%), Turkish (1.2%), Moroccan (1.9%), Ghanese (1.0%), Western (5.7%), non-western (4.4%). Mothers educational level was distributed as follows: 7.3% low (only primary school), 17.7% mid (high school or vocational training), and 75.1% high (university; in Dutch HBO and university). The mid educational level-group was overrepresented by non-Dutch participants, while the high educational level group was overrepresented by Dutch participants, and the low educational group was almost equally divided. When we compared our study sample to the larger population in the Amsterdam municipality in 2004, approximately 29.2% of the citizens were not born in the Netherlands. Approximately 20.6% of Amsterdam citizens followed higher education (van Zee et al., 2004). Although this comparison is not totally reliable because the total Amsterdam population also includes inhabitants that were not parents, this showed that our study was a relatively highly educated, ethnically more homogeneous sample. Our study sample did not differ significantly from the total group of participants at T2 (all participants at T2, including those who did not participate in the lab assessment) on educational level (*Χ*^2^ (4) = 6.34*, p* = 0.18), but did differ significantly on ethnicity (*Χ*^2^ (1) = 5.50*, p* = 0.02), with fewer non-Dutch children in our sample compared to the total sample (30%). Missing data from the individual variables ranged from 1.99% (trauma exposure) to 17.71% (inhibition 3).

### Procedure

All caregivers and participants older than 11 years (in some cases the participant just turned 12) gave informed consent. The information folders, letters and questionnaires were available in Dutch, English, and Turkish. Caregivers were asked to fill out questionnaires at home.

At T1, children completed four neuropsychological tasks individually on a laptop with a duration between 20 to 26 min. Trained instructors invited the children to perform the tasks individually in a quiet room at school. Instructors gave a verbal task instruction and demonstrated an example of the task. Then, the child performed a practice trial before starting the test trial of each single tasks (Guxens et al., 2016; Menting et al., 2018).

At T2, a total of 1006 children and their caregivers visited the research location and participated in both physical and mental health assessments. For the mental health assessments, children were interviewed face-to-face by trained psychologists and filled in questionnaires individually. The questionnaire that included items on executive functioning was send out by mail before the assessment day and caregivers filled this in at home. For more details on all measurement instruments and an overview of published research, see: https://www.amc.nl/web/abcd-studie-2.htm.

## Measures

### Childhood Trauma Exposure

Trained psychologists interviewed children with the life-events checklist (LEC) during T2. This semi-structured checklist is part of the Clinician Administered PTSD Scale for Children and Adolescents (CAPS-CA) (van Meijel et al. 2019; Pynoos et al., 2015). The checklist consists of 25 possible traumatic items, with five answer options; ‘*Happened to me’*, ‘*Witnessed it*’, ‘*Learned about it*’, ‘*Not sure*’ and ‘*Doesn’t apply*’. The LEC has good psychometric properties with a test–retest reliability of *r* = 0.82 and convergence validity with a mean kappa for all items of 0.61 (Gray et al., 2004). Besides questioning exposure to events, we also asked the child’s age during these events. After data collection and data entry, we rated the traumatic events based on the DSM–IV criteria. All events were rated by at least 2 out of 3 independent coders (RodK; JE; HB) and discrepancies were resolved by discussion with a third coder or expert panel.

We decided to include events that concerned participants themselves, their first degree relatives, or best friends only. Exceptions for this decision were cases of extreme violence such as victims of the attack on flight MH17 (in that case also teachers and friends were included). As a traumatic event is defined as one involving an actual or threatened death, serious injury or sexual violation, we decided that only severe accidents where an ambulance or hospital stay was needed, were included. For domestic emotional abuse we only included events that were extreme or caused structural safety issues for a longer period of time. As emotional abuse is mostly vaguely described by children (e.g. by mother yelled at me/called me names), we only included this as a traumatic event when the children reported that this happened more than once over a longer period of time. This was done by a “blind” expert panel of five experts in the field. In cases of discrepancies, consensus was reached by discussion. Approximately one-third of the children had been exposed to a traumatic event. Most traumatic events were severe accidents (27.5%) and victim of community violence (22.5%). Other events were disaster (1.6%), victim of domestic violence (7.8%), witness of domestic violence (7.5%), witness of community violence (5.9%), sexual assault (2.2%), death or injury of a loved one (6.7%), serious medical condition (14.9%), and other events (3.5%). Based on the interviews, we constructed two variables: traumatic events until the age of 5 (no / ≥ 1 event), and traumatic events between 6 and 12 years old (no, 1 event, ≥ 2 events). Twenty children (2.0%) did not participate in the interview.

### Executive Functions

Based on earlier research and theories, our approach was to focus on the conceptual unity underlying different aspects of executive functioning (Miyake & Friedman, 2000; Diamond, 2013). At T1, executive functioning was measured using subtests of the widely-used Amsterdam Neuropsychological Tasks (ANT) (Sonneville, 1999). The ANT is a computerized test battery that was performed in an individual setting at school in which the children performed the tasks pursuit, tracking, and response organization objects (ROO). The tasks have been shown to be sensitive to detection of neuropsychological problems in various samples and have good reliability and validity (De Sonneville et al., 2002; Rowbotham et al., 2009).The ROO task measures inhibitory control and cognitive flexibility and consists of three parts that increase in complexity. In part 1, children had to click the left mouse button when a green ball appeared on the left side of the screen and vice versa. In part 2, the tasks requires a click on the right mouse button when a red ball appeared on the left side of the screen and vice versa. In part 3, children had to follow these instructions based on the color of the ball that randomly alternated. A valid response was considered when a child clicked the correct button between 200 to 6000 ms after the stimulus was presented on the screen. Both the pursuit and tracking task measure visuomotor coordination. In the pursuit task, the child had to follow a mouse cursor on the screen that made a random trajectory with a constant speed of 10 mm/s, using their non-preferred hand and in the second part with their preferred hand. The tracking task is similar to the pursuit task, but in this task the mouse cursor follows a familiar and planned trajectory, which requires less executive demands.

The following outcome measures of these tasks were used to assess executive functions at age 5 (1) flexibility 1: mean reaction time compatible part 3 minus mean reaction time compatible part 1 in milliseconds, (2) flexibility 2: number of errors compatible part 3 minus mean reaction time compatible part 1, (3) flexibility 3: standard deviation right plus left hand compatible part 3 minus standard deviation right plus left hand compatible part 1 in milliseconds, (4) inhibition 1: mean reaction time incompatible part 2 minus mean reaction time compatible part 1 in milliseconds, (5) inhibition 2: number of errors incompatible part 2 minus number of errors number of errors compatible part 1, (6) inhibition 3: standard deviation right plus left hand compatible part 2 minus standard deviation right plus left hand compatible part 1 in milliseconds, and (7) motor flexibility: mean deviation overall pursuit – mean deviation overall tracking. The variables included were those that were most often reported focusing on inhibition and flexibility (Guxens et al., 2016; Menting et al., 2018). There was some missing data for these variables, as 15.51% of the children did not participate in the tasks. Furthermore, as the outcome is assumed to be unreliable when children outperform the difficult trials compared to the control trials of the tasks, negative contrast scores (in 0% to 14.5% of the cases) were recoded as invalid. To improve model convergence, we divided the values by constants to obtain variances with values between 1 and 10. A higher score on a variable corresponds with worse executive functioning.

We examined whether these variables could be modeled to load on one latent factor for executive functioning using the maximum likelihood with robust standard errors (MLR) estimator. Step 1 was to load the seven variables on one latent variable. This model had a poor model fit (*Χ*^2^ (14) = 462.69, *p* = 0.00, CFI = 0.69. RMSEA = 0.19). Modification indices showed – step by step – that the errors of inhibition 3 and flexibility 1, inhibition 1 and 3 should covary to improve the model. However, after adding the last error covariance, this model did not converge due to negative residual variance of inhibition 1. As this residual variance was non-significant, we could constrain the residual variance to zero and did not add the error covariance. We continued with adding step by step error covariances based on the modification indices between inhibition 3 and flexibility 3, flexibility 1 and 2, inhibition 2 with flexibility 2, inhibition 2 with flexibility 1, and inhibition 2 and 3. Inspection of the factor loadings indicated that inhibition 2 (0.11, *p* = 0.39) and flexibility 2 (0.17, *p* = 0.17) both had non-significant factor loadings on the latent variable. We excluded these variables (and their added error covariances) from the model. Therefore, the final measurement model included flexibility 1, flexibility 3, inhibition 1, inhibition 3, and motor flexibility as shown in Fig. 1. This model had excellent fit (*Χ*^2^ (4) = 0.38, *p* = 0.99, CFI = 1.00. RMSEA = 0.00), with standardized factor loadings ranging between 0.21 for motor flexibility to 0.76 for flexibility 3.

To measure executive functions at T2, 24 items (of a total of 75 items) of the Dutch parent version of the Behavior Rating Inventory for Executive Functioning (BRIEF) were used (Gioia et al., 2001; Huizinga & Smidts, 2009). The selected items cover eight subscales with three items each which were rated by caregivers. The questionnaire has eight subscales (inhibit, shift, emotional control, initiate, working memory, plan/organize, organization of materials and monitor) that are covered by the two indices Behavior Regulation Index (BRI) and Metacognition Index (MI). Statements such as “he/she struggles with finishing tasks” and “he/she gets upset in new situations” are scored on a three-point scale (*1* = *never, 2* = *sometimes, 3* = *often*). This means that a higher score on the subscales indicate poorer executive functioning. The questionnaire showed good psychometric properties in a sample of parents of 847 children with Cronbach’s alpha’s ranging from 0.78 to 0.96 (Huizinga & Smidts, 2009). Due to the long battery of questionnaires and to decrease the burden of participating in the research, we selected 24 items. In our study, the 24 items version of the BRIEF had an excellent reliability on item-level, as indicated by a Cronbach’s alpha of 0.91. Of the 1006 participants, 55 participants (5.5%) did not fill out the questionnaire. It is important to note that a higher score on these items corresponds with worse executive functioning.

We also examined whether these subscales could be modeled to load on one latent variable for executive functioning at age 12 using the MLR estimator. A model with all eight subscales of the BRIEF loading on one latent factor with error variances allowed to covary based on step-by-step modification indices, had an excellent model fit (*Χ*^2^ (7) = 8.79, *p* = 0.27, CFI = 0.99, RMSEA = 0.016). Error variances that covaried were: planning with initiate; emotion regulation with flexibility; inhibition with behavior evaluation, emotion regulation, and flexibility; behavior regulation with emotion regulation and flexibility; initiate with flexibility; organizing with working memory, flexibility and initiate. All standardized factor loadings were significant and in the expected direction and ranged from 0.31 for flexibility to 0.89 for working memory.

### Parenting Behavior

Parents reported on their parenting behavior by filling out the shortened version (32 items) of the Parental Styles and Dimensions Questionnaire (PSDQ) at T1. This scale was developed to investigate parenting styles using specific parenting practices that occur within the authoritative, authoritarian, and permissive parenting style. Due to the long battery of questionnaires and to decrease the burden of participating in the research, we used the shortened version of the questionnaire. This version consists of 15 items in the authoritative scale, 12 in the authoritarian scale, and 5 in the permissive parenting scale. In our study, items were rated on a four-point Likert type scale (*1* = *(almost) never; 2* = *once in a while; 3* = *often; 4* = *always)* for readability of the overall test battery. Parents responded on questions such as “I encourage my child to talk about its troubles”, “I punish by taking privileges away from my child with little if any explanation”, and “I spoil my child”. Scales were calculated by taking the sum score of the items within that scale. Psychometric properties of the 32-PSDQ have been investigated across various studies. Cronbach’s alphas ranged between 0.82 and 0.91 for authoritative, 0.67 and 0.86 for authoritarian, and 0.58 and 0.79 for permissive parenting. Good concurrent and predictive validity was also reported (Olivari et al., 2013). Although validity research on the shortened version is scarce, one study found its concurrent validity in relation to three other questionnaires to be sufficient (Topham et al., 2011). In our sample, we found Cronbach alpha’s of 0.82 for authoritative parenting, 0.71 for authoritarian parenting, and 0.59 for permissive parenting. As we found the reliability of the permissive parenting scale to be insufficient, we did not include these in our analyses. To assess the moderating role of parenting behavior, we used multi-group analyses. Therefore, the sample was split across the median for each of the parenting dimensions to create equal groups (authoritative parenting: 16; authoritarian parenting: 8).

### Prenatal Exposure

During their pregnancy, mothers reported on their cigarette, alcohol, and drugs intake. We combined these variables into one dichotomous variable. There was 0.4% missing data on this variable and 34.1% of the mothers reported on prenatal exposure of cigarettes, alcohol or drugs.

### Statistical Analyses

To answer our research questions, we performed structural equation modeling (SEM) using Mplus 7 (Muthén & Muthén, 2012) for analyses. Little’s Missing Completely at Random (MCAR) test was significant (*Χ*^2^ (292) = 541.30, *p* = 0.000), therefore we assumed that data were not missing completely at random. As missingness was not predictable from the dependent variables, we assumed that the data was Missing At Random (MAR) (Tabachnick & Fidell, 2013). We investigated whether cases with or without any missing data were significantly different from each other on all included variables using independent T-tests. Independent T-tests did not show significant differences between participants with missing data on T1 on measures of executive functioning at T2 nor the other way around. However, we found significant differences for all outcome measures of the ROO task and for authoritarian parenting behavior. This means, that on these variables, the mean scores were different for participants that had no missing data compared to participants that had missing information on one of the variables of interest. For prenatal substance abuse, we found significant differences between our sample and the total sample at birth (X^2^ (1) = 29.23, *p* = 0.00) as more mothers reported on prenatal substance use in our sample. For trauma exposure and executive functioning at age 12, we were not able to check whether our sample differed from the total sample of the birth cohort (starting at birth) as we only included participants that reported on trauma exposure at age 12. We checked normality of the data by investigating skewness and kurtosis and divided these statistics by their standard error. For all executive functioning variables, we found extreme positive skewness and kurtosis, which improved after dealing with univariate outliers. We modified the values to the closest observed value plus or minus one unit when z-scores exceeded ± 3.29, which resulted in an improved — but non-normal — distribution. We did not transform variables, as this would make interpretation merely impossible. We ran all models also with censored variables, and differences in models are reported when this was the case. We used the weighted least squares means and variance adjusted (WLSMV) estimator for the model analyses.

We constructed a longitudinal model as depicted in Fig. 1. After running our hypothesized model, we used multi-group analyses to investigate whether the link between trauma exposure and executive functioning was different across relatively low and high parenting behavior along the dimensions of authoritarian, authoritative, and permissive parenting. Model fit was assessed using comparative fit index (CFI; good model fit > 0.90) and Root Mean Square Error of Approximation (RMSEA; good model fit < 0.08) (Kline, 2005).

## Results

### Longitudinal Associations of Trauma and Executive Functioning

Means and standard deviations of independent, dependent and moderator variables are displayed in Table 1. To test our hypotheses, we ran the hypothesized model, which showed good model fit (*Χ*^2^ (72) = 136.54, *p* < 0.001, CFI = 0.98, RMSEA = 0.03), its coefficients are displayed in Fig. 1. With regard to concurrent associations, we found that trauma exposure was not associated with poorer executive functioning at age 5, but that this association was significant at age 12 (small effect). As expected for the longitudinal associations, trauma exposure was predictive of later poorer executive functioning (small effect) and later trauma exposure (small to moderate effect). However, the longitudinal association between executive functioning at age 5 and trauma exposure between age 6 and 12 was not significant.

**Table 1:** Means, standard deviations and percentages of independent, dependent and moderator variables

	Time 1			Time 2	
Variables					
	*n*	*%*		*n*	*%*
Trauma exposure					
No events	901	91.4	No events	671	68.1
1 event	78	7.9	1 event	233	23.6
≥ 2 events*	7	0.7	≥ 2 events	82	8.3
	*Mean*	*SD*		*Mean*	*SD*
Executive Functioning**					
Inhibition 1	369.67	209.04	Shift	4.27	1.38
Inhibition 2	2.21	3.51	Working memory	5.04	1.85
Inhibition 3	621.98	466.15	Initiate	5.18	1.53
Flexibility 1	772.18	326.24	Emotional control	4.10	1.32
Flexibility 2	2.87	4.14	Organization of materials	5.41	1.60
Flexibility 3	386.89	352.73	Monitor/evaluation	4.75	1.69
Motor flexibility	14.76	7.18	Plan/organize	5.37	1.69
			Inhibit	4.05	1.23
Permissive parenting***					
Low	6.97	0.88			
High	9.85	1.14			
Authoritarian parenting					
Low	15.33	1.29			
High	19.96	2.41			
Authoritative parenting					
Low	42.41	3.09			
High	50.71	3.04			

^*^Trauma exposure before age 5 was used as a dichotomous variable in the analyses. **Executive functioning at T1 is measured using the Response Objects Organization, Tracking and Pursuit tasks. Executive functioning at T2 is measured using the sum of three items of each subscale of the Behavior Rating Inventory of Executive Functioning (BRIEF). ***Parenting styles were split at the median for analyses into respectively low and high groups and was measured using a subset of the *PSDQ* Parental Styles and Dimensions Questionnaire

To control for educational level and prenatal exposure factors we included these factors in the model by regressing the variables at age 12 on the control variables and covary them with the variables at age 5. This model also had an excellent model fit (*Χ*^2^ (105) = 195.75, *p* < 0.001, CFI = 0.97, RMSEA = 0.03). After inclusion of these variables, the pattern of significant associations did not change, but educational level was significantly correlated with executive functioning at age 5. More specifically, a high maternal educational level was negatively associated with poorer executive functioning at age 5. This means that children of mothers with a high educational level had better executive functioning compared to children of mothers with a low or mid educational level. Coefficients, standard errors, and p-values are displayed in Table 2.

**Table 2:** Coefficients, standardized coefficients, standard errors and p-values of model with control variables

	B	β	S.E	*p*-value
Executive functioning age 5 → executive functioning age 12	0.044	0.060^a^	0.041	0.148
Trauma exposure before age 5 → executive functioning age 12	0.188	0.121	0.036	0.002
Executive functioning age 5 → trauma exposure between age 6 and 12	-0.070	-0.042	0.045	0.347
Trauma exposure before age 5 → trauma exposure between age 6 and 12	1.515	0.425	0.031	0.000
Executive functioning age 5 ↔ trauma exposure before age 5	-0.007	-0.041	0.041	0.325
Executive functioning age 12 ↔ trauma exposure between age 6 and 12	0.052	0.134	0.044	0.005
Prenatal drug exposure ↔ executive functioning at age 5	-0.010	-0.035	0.039	0.366
Prenatal drug exposure ↔ trauma exposure before age 5	-0.001	-0.007	0.032	0.828
Prenatal drug exposure → executive functioning at age 12	-0.010	-0.010	0.036	0.773
Prenatal drug exposure → trauma exposure between age 6 and 12	0.065	0.031	0.035	0.386
Mid maternal educational level ↔ executive functioning at age 5	0.015	0.065	0.037	0.074
Mid maternal educational level ↔ trauma exposure before age 5	0.006	0.059	0.030	0.054
Mid maternal educational level → executive functioning at age 12	-0.078	-0.068	0.066	0.305
Mid maternal educational level → trauma exposure between age 6 and 12	-0.268	-0.102	0.071	0.149
High maternal educational level ↔ executive functioning at age 5	-0.023	-0.088	0.036	0.014
High maternal educational level ↔ trauma exposure before age 5	-0.005	-0.040	0.032	0.217
High maternal educational level → executive functioning at age 12	-0.120	-0.119	0.066	0.079
High maternal educational level → trauma exposure between age 6 and 12	-0.304	-0.131	0.071	0.064

^a^Following guidelines, all estimated are standardized using STDYX standardization in Mplus, expect for the longitudinal association between executive functioning at age 5 and age 12, then STD standardization is used. For analyses purposes, dummy variables were made for maternal educational level in which low maternal educational level was the reference category

### Moderating Role of Parenting Behavior

To investigate the moderating effects of parenting behavior, we performed three separate multi-group analyses on the final model that controlled for prenatal exposures and maternal educational level. First, we tested a model with all parameters constrained across groups against a model with the hypothesized associations freed across groups using the DIFFTEST option in Mplus. For authoritative (Δχ^2^ = 8.94, Δ df = 6, *p* = 0.18) and authoritarian parenting (Δχ^2^ = 3.25, Δ df = 6, *p* = 0.78) no significant group differences were found. This means that paths in the model were not different for parents with a relatively high or low score on the subscales of authoritative and authoritarian parenting.

## Discussion

In the present study, we analyzed concurrent and longitudinal associations between trauma exposure and executive functioning in a birth cohort using structural equation modeling. Our primary aim was to distinguish the direction of relationships between trauma exposure and executive functioning in children. When we gain more insights in the direction of this relationship we can offer some implications for clinical practice and further research. Our results demonstrated that after controlling for prenatal drug exposure and maternal educational level, early trauma exposure was indeed predictive of poorer executive functioning later. Although we had hypothesized that early poorer executive functioning could also be a risk factor for later trauma exposure, we did not find such an association. Also, we did not observe evidence to suggest that maternal parenting behavior moderates the longitudinal association between trauma exposure and subsequent executive functioning. We did, however, find that, while trauma exposure before age 5 was not associated with executive functioning at age 5 it was predictive of poorer executive functioning at age 12. Specifically, we can conclude that early trauma exposure does indeed predict parent-reported executive functioning, but we are not able to draw conclusions whether this would also be the case for objective executive functioning measured by neuropsychological tasks. Moreover, executive functioning at age 5 did not seem to be associated with subsequent trauma exposure.

This study’s findings are in line with earlier research that did not control for pre-existing executive functioning in trauma-exposed youth growing up in a deprived institutional setting (Bos et al., 2009; Jennifer Martin. McDermott et al., 2013). The findings are also in line with studies that did control for earlier cognitive functioning (Enlow et al., 2012; Geoffroy et al., 2016), but did not examine executive functioning specifically. The fact that we replicate these earlier findings is of importance given that we observed these associations even when the children in our sample experienced relatively “less severe” trauma exposure (mostly severe accidents rather than extreme neglect or maltreatment) and had a relatively high socio-economic status as compared to the children participating in these earlier studies.

Although not a main aim of our study, it is interesting to note that early trauma exposure was not only predictive of later poorer executive functioning, but also predictive of later trauma exposure. Longitudinal co-occurrence of adverse childhood events has previously been found (Green et al., 2010; McLaughlin et al., 2012). Based on cumulative risk theory, accumulation of trauma exposure has been found to predict more long term problems including mental health problems (McLaughlin & Sheridan, 2016). The fact that we also found this accumulation of trauma exposure across childhood, even in a relatively highly educated community sample, underscores the importance of population-wide early screening, prevention, and intervention efforts.

In our study, we did not observe a significant longitudinal association between executive functioning at age 5 and 12. Previous research indicated that, even measured at the same time, there is often a relatively weak link between executive functions measured by tasks and measured by self-report questionnaires (e.g. Kenworthy et al., 2008; Silver, 2014; Toplak et al., 2013). In our study too, the discrepant measurement strategies (T1: tasks; T2: questionnaire) across the waves could have led to this non-significant association. An alternative explanation for the fact that we did not find a longitudinal association for executive functioning could be that we used different measures of executive functioning at T1 (core executive functions are the key components) and T2 (core executive functions and higher order executive functions are combined).

Additionally, although we did find a significant covariance between trauma exposure and executive functioning at age 12, we did not find a significant covariance at age 5. Based on an earlier review and a meta-analysis, an association between trauma exposure and executive functioning was expected (Malarbi et al., 2017; Op den Kelder et al., 2018). As the concurrent link between trauma exposure and executive functioning at age 5 was not significant, this supports the finding that trauma exposure leads to later executive functioning problems and that this process takes time. Additionally, it supports our finding that earlier levels of executive functioning does not predict later trauma exposure. However, it is still possible that a concurrent link between trauma exposure and executive functioning becomes more visible later in childhood. The majority of research is based on children aged above eight years old, which results in a limited understanding of the relationship between trauma exposure and executive functioning in younger children. Our results indicate that the effect of early trauma exposure may not yet be visible on neuropsychological tasks in five year old children.

### Parenting Behavior as a Moderator

The secondary aim was to investigate the moderating role of maternal parenting behavior in the association between trauma exposure and executive functioning. We found no moderating effect of maternal parenting behavior in the associations between trauma exposure and executive functioning. This means that overall maternal parenting behavior did not buffer or exacerbate the longitudinal relationship between trauma exposure before preschool and executive functioning and trauma exposure later in life. As our study is the first to examine this association, more research is necessary to draw firm conclusions. Possibly, trauma exposure could have such an overridingly strong effect, that maternal parenting behavior does not come out as a significant moderator in our analyses. It could be that there are other protective factors that outweigh the buffering effect of authoritative parenting behavior such as social support or secure attachment style. Alternatively, accumulation of protective factors is needed for resilience against trauma exposure in early childhood (Sattler & Font, 2018). In other words, although we did not find moderating effects of maternal parenting behavior, it does not mean that parents cannot support their children after trauma exposure and thereby diminish the consequences of trauma exposure. It could be that some specific parenting behaviors such as providing structure, warmth and calmness, and regulatory practices (that are not specified in an overall authoritative parenting style) do help children after they have been exposed to a traumatic event.

### Strengths & Limitations

Our study has several strengths. First, our study was based on a relatively large community sample with a longitudinal design. This increases power and makes it possible to use sophisticated statistical methods such as structural equation modeling. Second, we used a multi-informant and multi-method approach which reduces mono-method bias. However, several limitations are also important to take into account. Although the ABCD birth cohort aimed to be as ethnically diverse as the Amsterdam community, compared to the Amsterdam community, the sample included a relatively high number of Dutch children from parents with higher educational levels. This is a limitation as our results are not directly generalizable to the Amsterdam community nor to samples with a lower socio-economic status. However, as aforementioned, the fact we still observe the longitudinal association between trauma exposure and executive functioning seven year later in this relatively healthy community sample is a strong indicator for the strength of this relationship.

Second, we were limited by the retrospective assessment of trauma exposure at age 12. Retrospective reports may be biased due to difficulties remembering (correctly). We aimed to improve the retrospective assessment as much as possible by using face-to-face interviews with detailed questions about life events by trained developmental psychologists, which enabled us to ask specifically about facts regarding the traumatic events.

Third, it is important to realize that the use of a median split for our moderator reduced variance, which may have impacted the results. We cannot rule out that a significant moderation effect of parenting would occur at a very high or very low level of our moderator. At the same time, as there are no clinical cut-offs for our parenting measure, it is difficult to argue for a choice of a cut-off. A median split ensures comparable sample size in both groups. However, future research using different models, may be able to include a continuous moderator for parenting behavior and examine this possibility.

Fourth, as we used different assessment methods of executive functioning we can only interpret our findings on that specific assessment type (tasks or questionnaire). Therefore, we are limited in differentiating between age- and measurement-method effects in our study. Finally, the questionnaire that was used for measuring maternal parenting behavior had a good reliability for authoritarian and authoritative parenting, but not sufficient reliability for permissive parenting (both in previous research and in our sample). This made it impossible to investigate the moderating role of maternal permissive parenting behavior. Notwithstanding our study’s limitations, our study is the first to distinguish the two possible predictive relationships between trauma exposure and executive functioning, in a well-powered, large sample of families from the general population, using a sophisticated, SEM based analytical strategy.

In this study, we modelled executive functioning with one latent factor because we were interested in the common component underlying executive functioning in relation to trauma exposure. However, the fact that the errors of our executive functioning indicators were correlated might indicate that executive functioning in our sample consisted of subfactors. Although there are different findings in previous research in terms of unity/diversity of executive functioning in children, based on a recent review most evidence is found for unidimensionality of executive functioning in young children and adolescents (Karr et al., 2018). As a very recent study argued that the chosen measurement model for executive functioning might impact interpretation of results (Camerota et al., 2020), future studies should investigate whether modeling a latent factor structure, subcomponents or a composite score are differentially related to trauma exposure than the common component we modeled here.

### Future Research

Although we found a predictive association between early trauma exposure and later executive functioning, our results do not provide insight on the mechanisms that may underlie this association. Resilience and vulnerability after trauma exposure are thought to arise from complex interactions between various systems such as genetics, structure and functioning of the brain, cognition, social environment, endocrinology, and the immune system (Ioannidis et al., 2020). It is possible that long-lasting neurobiological changes play a role in these complex interactions. At the same time it could be that trauma-exposed children suffer from posttraumatic stress symptoms which in turn leads to poorer executive functions. In this case, it could be possible that poorer executive functioning is more temporary, and trauma-focused treatment may alleviate problems in executive functioning. There is very little research on this topic, but one study among fifteen women found medium-sized improvements on cognitive flexibility and planning three months after the start of trauma-focused treatment (Walter et al., 2010). Future research should therefore try to examine how trauma exposure results in executive functioning problems in children and whether these problems are alleviated after trauma treatment. Future research should also focus on a possible critical time frame and accumulation of trauma exposure in order to investigate the neurobiological effects of trauma exposure in childhood. More knowledge on these mechanisms makes it possible to develop and implement prevention and intervention programs for trauma-exposed youth. Additionally, as we focused on the conceptual unity of executive functioning while taking into account its differentiability in our latent factor model, it would be interesting to investigate executive functioning more specifically and thereby focus on specific pathways of working memory, inhibition, and flexibility.

## Conclusion

Results of our study do not support the notion that associations between trauma exposure and executive functioning can be explained by pre-existing executive functioning problems acting as a risk factor for trauma exposure. Rather, our findings indicate that trauma exposure impacts developing executive functioning of the child. These findings result in a few clinical and practical implications. First, as our study suggest that early trauma exposure predicts executive functioning and further trauma exposure over a course of seven years, youth health services for young children could play an important role in recognizing trauma exposure. Based on earlier research we know that an accumulation of trauma exposure in childhood leads to a higher risk of development of posttraumatic stress and other health issues (Green et al., 2010; McLaughlin et al., 2012). Additionally, we are also aware of the relatively high impact of problems in executive functioning in daily life (Diamond, 2013). We urge general mental and physical health practice, also because of protective reasons, to include specific questions about trauma exposure in their standard protocols in both early childhood as well as during early and late adolescence. Second, as later executive functioning is predicted by early trauma exposure, it is important to include executive functioning in the assessment of traumatized youth in clinical practice.

In conclusion, our results suggest that trauma exposure before age 5 is predictive of poorer parent-reported executive functioning and trauma exposure at age 12. This longitudinal association could not be explained by pre-existing poor executive functioning (measured by neuropsychological tasks), as executive functioning at age 5 was not associated with trauma exposure before age 5, and was also not predictive of trauma exposure up to age 12. Our findings are based on a community sample with relatively “mild” trauma exposure and a relatively high socio-economic-status; which implies that even under these circumstances, these mechanisms are at work. We would like to suggest that clinical practice takes this into account in the implementation of prevention and intervention programs after trauma exposure. The scientific field should aim to replicate our findings in different samples, using multiple measurement instruments for executive functioning.

## Data Availability

Data are from the ABCD cohort study. These data are not available for a public repository for ethical reasons. These data restrictions were specifically imposed by the medical ethics committee of the Academic Medical Centre in Amsterdam. However, the ABCD study can be contacted for requests to access the data (abcd@amc.uva.nl).
