# Correction to: Longitudinal Associations Between Trauma Exposure and Executive Functions in Children: Findings from a Dutch Birth Cohort Study

**DOI:** 10.1007/s10802-022-00984-4

**Published:** 2022-11-04

**Authors:** R. Op den Kelder, A. L. Van den Akker, J. B. M. Ensink, H. M. Geurts, G. Overbeek, S. R. de Rooij, T. G. M. Vrijkotte, R. J. L. Lindauer

**Affiliations:** 1 Research Institute of Child Development and Education, University of Amsterdam, Amsterdam, The Netherlands; 2 Levvel Academic Center for Child and Adolescent Psychiatry, Amsterdam, The Netherlands; 3 Research Institute of Child Development and Education/Research Priority Area YIELD, University of Amsterdam, Amsterdam, The Netherlands; 4 Department of Child and Adolescent Psychiatry, Amsterdam UMC, Location AMC, University of Amsterdam, Amsterdam, The Netherlands; 5 Department of Psychology (Brain and Cognition)/ Research Priority Area YIELD, University of Amsterdam, Amsterdam, The Netherlands; 6 Department of Public Health, Amsterdam UMC, Amsterdam Public Health Research Institute, University of Amsterdam, Amsterdam, The Netherlands; 7 Department of Clinical Epidemiology, Biostatistics and Bio-Informatics, Amsterdam UMC, University of Amsterdam, Amsterdam, The Netherlands

**Correction to: Research on Child and Adolescent Psychopathology (2021) 50:295–308.**

10.1007/s10802-021-00847-4.

The article “Longitudinal Associations Between Trauma Exposure and Executive Functions in Children: Findings from a Dutch Birth Cohort Study”, was originally published electronically on the publisher’s internet portal on 5 September 2022 with error on descriptive information found in ‘Study Population’ and ‘Childhood Trauma Exposure’ subsections.

Additionally, in Table 1, the number for exposure to one traumatic event at T1 should be 78 and not 785 as originally displayed in the paper.

The original article has been corrected.

